# CD133-Guided RNA Nanoparticle Delivery of FTO siRNA Impairs Leukemia Resistance to Tyrosine Kinase Inhibitor Therapy

**DOI:** 10.59566/isrnn.2025.0201e

**Published:** 2025-04

**Authors:** Huiqin Bian, Changli Zhou, Hiroaki Koyama, Wen Gao, Sicheng Bian, Tao Cheng, Xiaonan Han, William Tse, Alexander Miron, Shujun Liu

**Affiliations:** 1 Department of Medicine, The MetroHealth System, Case Western Reserve University, 2500 Metro Health Drive, Cleveland, OH 44109, USA;; 2 Gene and Cell Therapy Institute (GCTI), The MetroHealth System, Case Western Reserve University, 2500 Metro Health Dr, Cleveland, OH, USA;; 3 Department of Genetics and Genome Sciences, Case Western Reserve University Medical School, 2109 Adelbert Road, Cleveland, OH 44106, USA

**Keywords:** FTO, m6A, Leukemia, drug resistance, tyrosine kinase inhibitors, leukemia stem cell, RNA nanoparticles, CD133, colonies, spheroids, Heterogeneity

## Abstract

Despite the initial responses to the tyrosine kinase inhibitor (TKI) for cancer therapy, many patients often relapse with no curative regimens available. Further, the ability to target therapeutic agents to cancer cells with appropriate doses remains challenging in the clinic, especially for leukemia. Here, we show that naïve CML cells are dynamically heterogeneous in colony formation. Larger clones expand while smaller ones diminish and eventually disappear. Compared to resistant cells, parental populations, including CD44+ stem cells, form a greater number of larger, solid spheroids. Upregulation of fat mass and obesity associated protein (FTO), an RNA *N*^*6*^-methyladenosine demethylase, and stem cell markers (e.g., CD44, CD133, CD25) is more obvious in resistant cells compared to parental cells. FTO inhibitors (e.g., CS1, FB23-2) appreciably impair the growth of resistant cells either alone or in combination with nilotinib. FTO protein expression is unexpectedly upregulated by CS1 or FB23-2 treatment in multiple leukemia cell lines. We then constructed RNA nanoparticles encapsulating FTO siRNAs and conjugated with anti-CD133 RNA aptamers. We showed that, compared to negative control, these nanoparticles were taken up much more efficiently by resistant cells that highly express CD133. Treatment with the CD133-guided FTO siRNA nanoparticles efficiently silenced FTO expression in resistant cells, which leads to a significant reduction in their colony and spheroid formation. These findings offer new insights into cancer drug resistance and advance the application of RNA nanotechnology for treating leukemia. The research provides a foundation for developing novel, targeted therapies for resistant leukemia.

## GRAPHICAL ABSTRACT

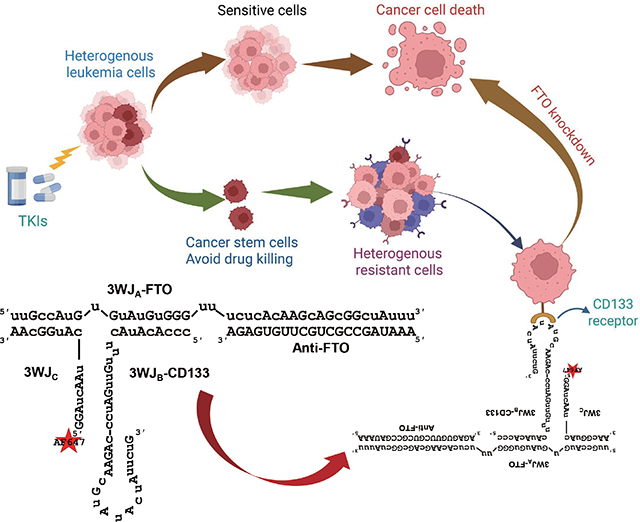

## INTRODUCTION

Leukemia is an aggressive malignancy frequently associated with the constitutively active tyrosine kinases (TKs).^1–4^ Multiple TK inhibitors (TKIs) in clinical use have revolutionized leukemia treatment.^5–8^ However, a significant proportion of patients experience disease recurrence due to drug resistance,^9–12^ representing a major hurdle for successful leukemia treatment.^13–19^ Further, among those patients (30–50% in CML) who achieve complete molecular remission, 30–50% of patients must take “pricey and toxic” lifelong TKIs,^20–23^ which is associated with severe side effects and huge financial burden. The most cited genetic event is the acquired drug-resistance mutations that impair drug binding or bypasses inhibited TK signaling.^15,24^ However, genetic selection does not fully explain the development of therapeutic resistance. Evidence is emerging that non-genetic mechanisms of resistance are important in therapeutic failure.^25–28^ But gaps concerning the key epigenetic events in developing and sustaining TKI resistance still exist.

Abundance of *N*^*6*^-methyladenosine (m^6^A), the most common modification in RNA,^29–35^ is determined by multiple factors including fat mass and obesity associated gene (FTO), a major RNA m^6^A eraser.^36–39^ While its key role in cancer pathogenesis is established,^40–44^ the knowledge about its function in drug resistance is limited. Our prior study^45^ suggests that the dysregulated FTO/m^6^A axis is a novel pathway for leukemia cells to avoid TKI-induced cell death. This is because leukemia cells with FTO upregulation and m^6^A demethylation exhibit more TKI tolerance and higher growth rates. In contrast, FTO inactivation by siRNAs or small molecule inhibitors^46,47^ restores m^6^A methylation and renders resistant cells sensitive to TKIs.^45^ Further, proteolysis targeting chimeras (PROTACs), which target proteasomal degradation of disease-related proteins (e.g., FTO),^48^ show promise in treating cancer patients, collectively, supporting FTO as a new therapeutic target to overcome TKI resistance. However, *in vivo* use of these FTO inhibitory molecules cause toxicity and off-target effects.^46,49^ For example, the poor water solubility, low cellular permeability, and off-target side effects of most PROTACs and siRNAs prevent them from benefiting cancer patients,^50^ calling for more efficient delivery strategies.

RNA nanoparticles^51–54^ are distinct from conventional nanomaterials.^55–59^ The RNA nanoparticles derived from phi29pRNA-3WJ^60–62^ can be multivalent vectors to carry RNAi, RNA aptamers, or chemical ligands targeting cancer cell surface receptors (e.g., CD133, EGFR).^63–67^ These chemically modified, RNase-resistant, and thermodynamically stable RNA nanoparticles block tumor growth in murine models without observed toxicity.^68,69^ Over 5% of targeted RNA nanoparticles accumulate in tumors with little accumulation in vital organs, compared to only 0.7% of other nanoplatforms.^70,71^ Further, leukemia stem cells (LSCs) are a myeloproliferative disorder of stem cell origin. LSCs are known to be resistant to TKIs,^72–75^ and persist in patients on long-term therapy, where they fuel TKI resistance, empowering relapse and disease progression.^12,72^ For example, the persistent CD26+ LSCs were identified in CML patients with molecular response, receiving TKI treatment or after TKI discontinuation.^76^ CML LSCs have an aberrant expression of markers (CD25, CD44, CD117/KIT, CD133) even when the patients are diagnosed.^77–79^ Dysregulation of these markers predicts drug resistance.^77–79^ In this project, we found that LSC markers are highly expressed in TKI resistant cells through a FTO/m^6^A axis. We loaded RNA nanoparticles with CD133 ligands, which specifically deliver FTO siRNAs to drug-resistant K562 cells by avoiding endosomal trapping in cancer cells. We show that FTO siRNAs delivered by RNA nanoparticles efficiently knock down FTO gene expression impairing the formation of colony and spheroids, suggesting that knocking down FTO by RNA nanoparticle delivered siRNAs has therapeutic potential in resistant leukemia.

## RESULTS

### The heterogeneous states of clones in naïve leukemia cells are intrinsic, transient, and dynamic

**Fig. 1.**
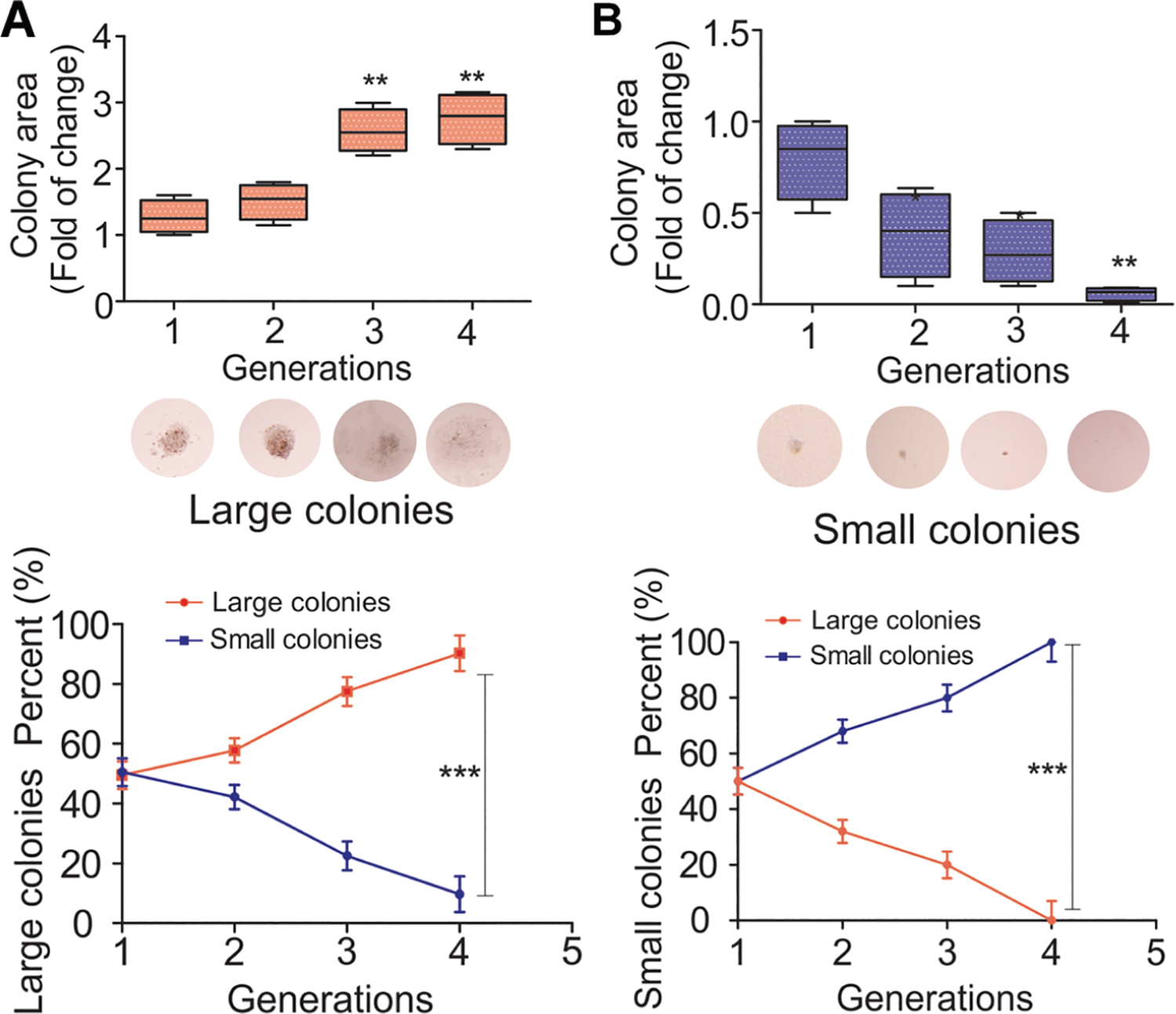
The formation and maintenance of heterogeneous leukemia clones is dynamic. (A-B) Repetitive replating of single K562 colonies demonstrates the regeneration of clonal heterogeneity. Naïve, unselected K562 CML cells were subjected to an initial colony-forming assay (CFA) by seeding at a low density. After 10 days of incubation, the resulting colonies were categorized by size. Representative large clones (A) or small clones (B) were individually isolated. The cells from each isolated clone were then dissociated into a single-cell suspension and re-plated. This process was repeated for 10 successive rounds, with each generation lasting 10 days. The data show that both the large and small isolated clones regenerate the full heterogeneous size distribution of colonies upon replating. The area cutoff is 848 μm^2^; cell number cutoff is 50. Data represents the mean ±SD of triplicate experiments. **P <0.01, ***P <0.001.

While the BCR/ABL oncogene is sufficient to drive CML suggesting genetic homogeneity, the disease also exhibits epigenetic and phenotypic heterogeneity. This is evidenced by distinct epitranscriptomic profiles (FTO/m^6^A) found in both naive and drug-resistant cell populations, and by the diversified FTO/m^6^A signatures observed in naive cells that form colonies of varying sizes.^45^ To determine whether this heterogeneity is static or dynamic, naïve and unselected K562 cells were analyzed using a colony-forming assays^45,80–82^ without TKI exposure. The cells were seeded at low density to allow individual cells to grow into separable colonies. As shown in Fig. 1A, 1B, the colonies varied in size (classified into large or small; the area cutoff is 848 μm^2^, the number cutoff is 50) and morphology, reflecting the cellular heterogeneity of the initial population, extending our previous findings^45^ that naïve leukemia cells are phenotypically heterogeneous.

To investigate the heritability and maintenance of clonal characteristics, individual large or small colonies were isolated. Each isolated clone was then subjected to 10 rounds of serial replating. In this procedure, cells from an individual colony were harvested, counted, and re-plated at a low density to initiate the next generation of colonies. Each generation of replating spanned 10 days, after which new colonies were analyzed for size and replated again. This process was repeated for 10 consecutive generations, allowing the tracking of clonal fidelity and any shifts in the colony size distribution over time. For each plating, we measured the number and size of the colonies. We found that larger clones have a higher potential to form and retain as large clones, while smaller clones retain and continuously form an increased number of smaller clones, then decay till disappear after 10 passages. These results indicate that K562 leukemia clones are not static, but instead are composed of a dynamic hierarchy of cells that can regenerate the original diversity of colony phenotypes over multiple generations.

### Drug-resistant cell-derived spheroids show altered stem cell activity compared to parent cell-derived spheroids

**Fig. 2.**
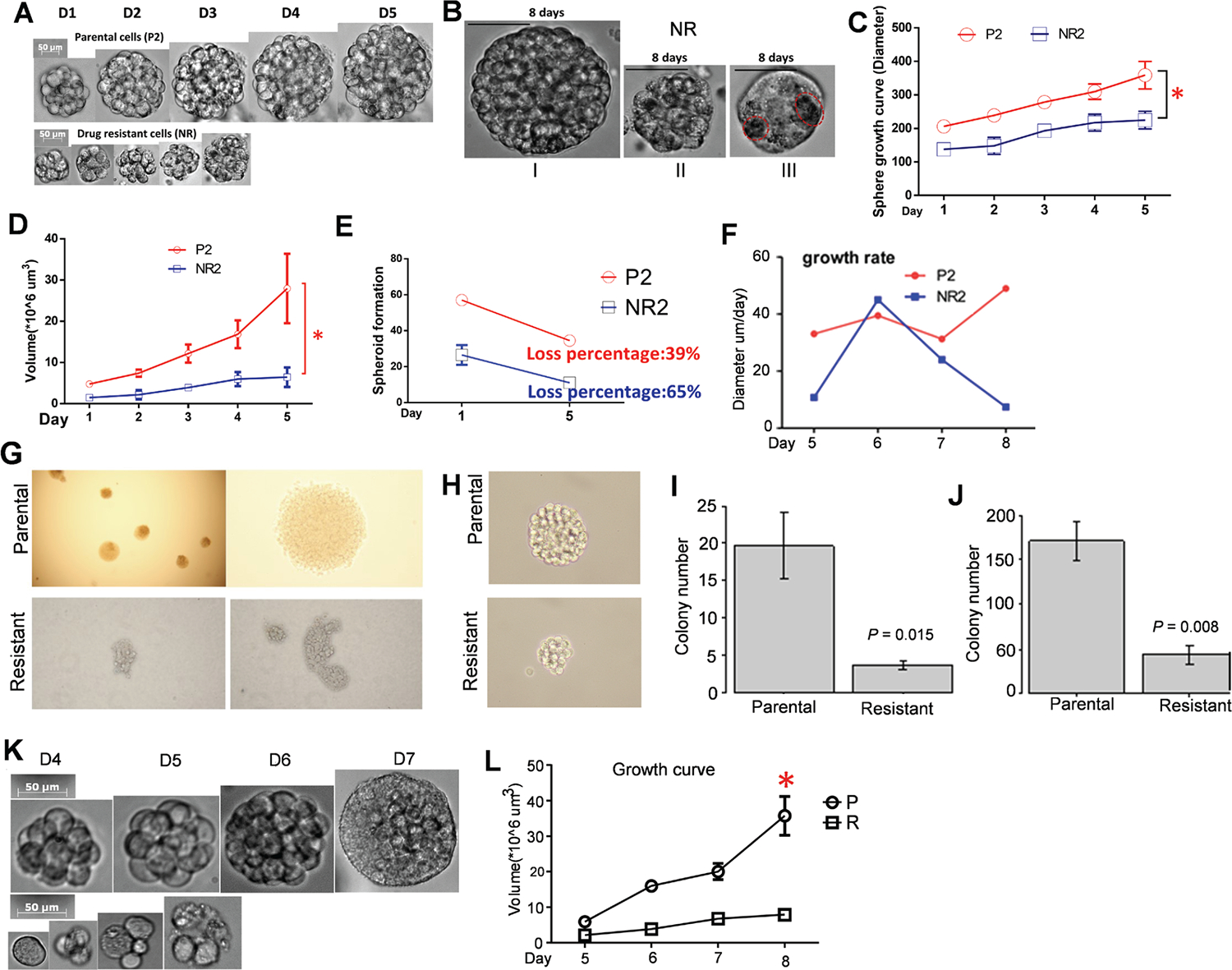
Generation and characterization of spheres from drug resistant (NR) or parental (P2) cells. (A) Sphere images were captured for 5 days, bar = 50 μm. (B) P2 and NR spheres were classified into three morphologies: (I) spheres with solid lumen, (II) small, tightened spheres, and (III) semi-spheroids with lumens. Open red circles indicate several lumens. (C) Growth curves show that NR spheres have smaller diameters than P2 spheres. (D) Volume curves demonstrate that NR spheres are smaller than P2 spheres. (E) Quantification of spheroid lumen shows that 65 % of NR2 spheres exhibit no lumens while 39% of P2 spheres have no lumens. (F) The rate of increased diameter shows that diameters in daily growth are reduced by 4 days in the NR-derived spheres compared to P2 spheres. All results are expressed as mean ±SEM from five independent experiments. (G) Representative images of a colony formation assay showing the sizes and numbers of colonies formed by CD44+ cells. (H) Representative images from Matrigel Matrix analysis, illustrating the shapes and sizes of colonies derived from CD44+ cells. (I-J) Graphs showing the number of colonies (I) or spheres (J). (K-L) Cellular heterogeneity of drug resistance requires stem cells. CD44+ cells were isolated from parental or resistant cells and subjected to spheroid forming assays. Images were captured daily by the Live cell system. The spheroids were differentiated for 7 days (K). The size of spheroids was measured daily and expressed as volume (mm^3^) to generate the growth curves (L). Results were expressed as mean ±SEM, *P <0.05. P/P2, parental cells; R/NR2, nilotinib resistant cells; D1……D7, day 1 ……day 7.

Our previous findings demonstrated that larger clones in naïve cells have higher drug tolerance than smaller clones.^45^ This subtle, initial difference in drug sensitivity serves as the foundation for developing drug-resistant populations and colonies, but the overall contribution of clonal heterogeneity to this development remains undetermined. To investigate this, we established drug-resistant cell populations by chronically exposing CML K562 cells to physiological doses of the TKI, nilotinib. 3D spheroid model, whose architecture mimics the tumor microenvironment (TME), offers a more physiologically relevant system than traditional 2D cultures by incorporating crucial elements like gradients of oxygen and nutrient.^83^ This heterogeneous environment, combined with drug selection, exerts a powerful selective pressure on different leukemia cell subpopulations, favoring those adapted to conditions like hypoxia. However, the application of 3D spheroid model in leukemia study is underexplored. To address this gap, we conducted spheroid forming assays on parental (P2) and resistant (NR) K562 cells. P2 and NR cells were embedded with growth factor-free Matrigel and differentiated into sphere-like structures for 10 days, during which the media were changed once per 4 days. Morphology and stem cell activity were defined as growth curve, volume, lumen numbers, and rate of growth speed from day 5. We found that P2 and NR cells form distinctive spheroids (Fig. 2A, 2B). NR spheres had significantly smaller diameters (Fig. 2C) and sizes (Fig. 2D) than P2 spheres. About 65 % of NR2 spheres exhibited no lumens, while 39% of P2 spheres had no lumens (Fig. 2E). Diameters in daily growth are reduced by 4 days in the NR-derived spheres compared to P2 spheres (Fig. 2F), highlighting the inherent growth differences of these selected clones. Collectively, findings from our spheroid model demonstrate how drug selection on clonal heterogeneity fundamentally alters leukemia cell behavior. These distinct biological adaptations lead to, even in an *in vivo* context, altered leukemia-TME crosstalk through paracrine factors, extracellular matrix remodeling, and metabolic changes.

The role of stem cells in forming tumoroids is well-documented,^84^ but these studies are limited in the context of leukemia. To investigate the effects of TKIs on LSCs, we performed colony- and spheroid-forming assays on CD44+ cells sorted from parental and nilotinib resistant cell populations. CD44+ LSCs from the resistant populations consistently formed smaller, sparse, loose, and irregular colonies and spheroids (Fig. 2G, 2I), suggesting a higher degree of heterogeneity within these cells.^85^ In contrast, colonies and spheroids derived from parental CD44+ LSCs were larger, more numerous, and had a regular, hollow structure (Fig. 2H, 2J). Notably, the CD44+ LSCs sorted from drug-resistant populations showed more dynamic and rapid spheroid formation and growth (Fig. 2K, 2L). These results suggest that chronic drug exposure modifies LSC cellular behaviors, potentially influencing their capacity to avoid drug killing.

### FTO protein expression is unexpectedly increased in vehicle vs FTO inhibitors-treated cells

**Fig. 3.**
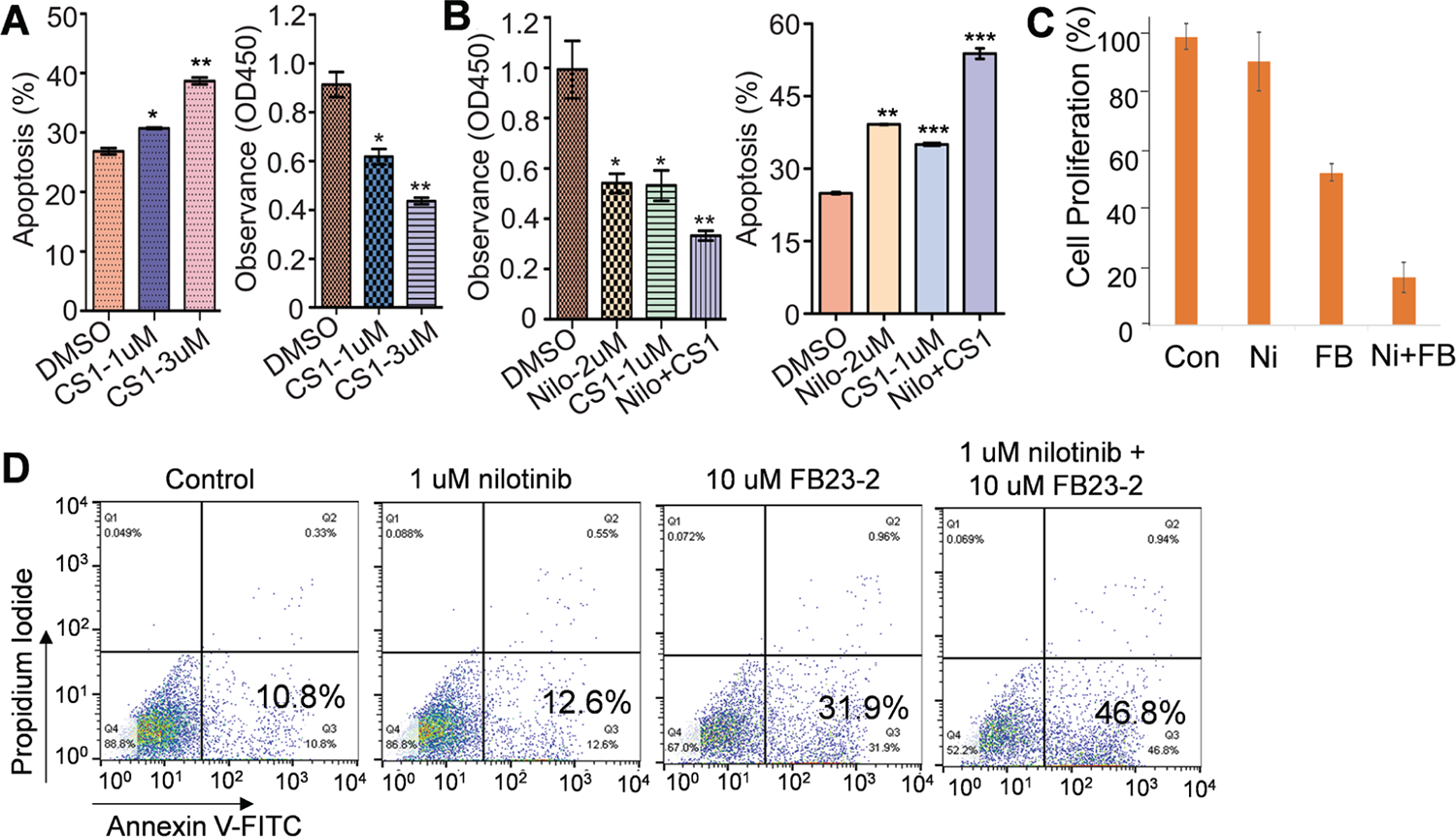
The effects of FTO inhibitors on nilotinib-resistant cell growth. (A-B) Nilotinib-resistant K562 cells were treated with either CS1 (a selective FTO inhibitor; 1 or 3 μM) alone (A) or in combination with nilotinib (B) for 48 hours and subjected to CCK-8 for cell growth or flow cytometry for cell apoptosis. (C-D) Nilotinib-resistant K562 cells were treated with either nilotinib (1 μM), FB23-2 (a selective FTO inhibitor; 10 μM), or the combination for 48 hours. Cell viability was measured using a CCK8 assay (C), and flow cytometry was used to analyze cell apoptosis (D). Data in CCK8 assays represents two independent experiments, each with three replicates. Con, control; Ni, nilotinib; FB, FB23-2; Nilo/Ni, nilotinib; FB, FB23-2; *P <0.05, **P <0.01, ***P <0.001.

**Fig. 4.**
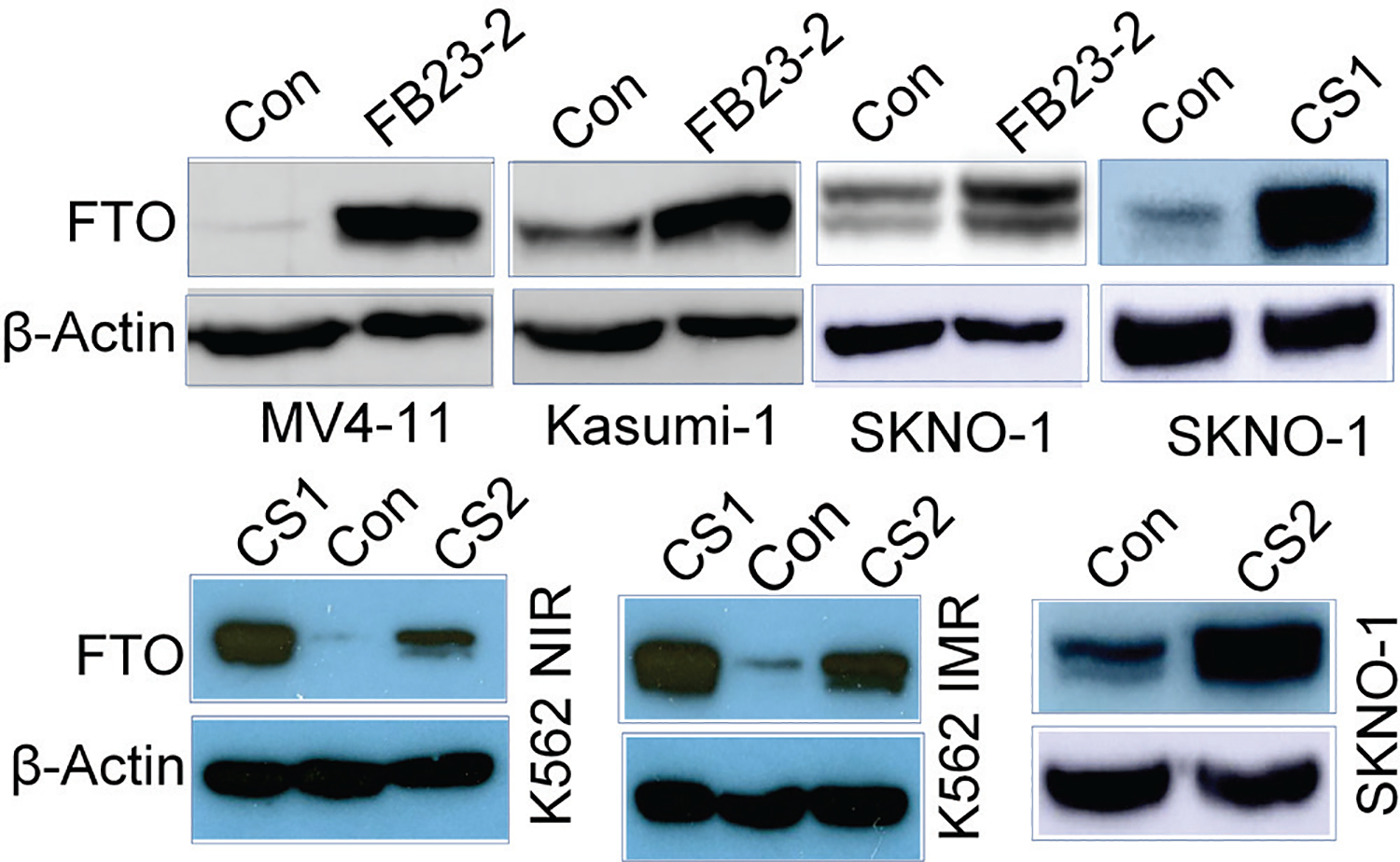
FTO inhibitors induce the upregulation of FTO protein expression. Leukemia cells Kasumi-1 (AML, with AML1/ETO translocation and an activating KIT mutation), SKNO-1 (AML, with AML1/ETO translocation and an activating KIT mutation), MV4–11 (AML, with AF-4/FEL translocation and activating FLT3 mutation) and K562 (CML, with BCR/ABL translocation and activating ABL kinase) were treated with FB23-2 (10 μM), CS1 (1 μM) or CS2 (1 μM) for 48 hours. The FTO protein expression was determined by Western blot. Data are representative of three independent experiments. NIR, nilotinib resistance; IMA: Imatinib resistance; Con, Control.

Evidence from our studies and others shows that an FTO/m^6^A axis mediates the development of drug resistance in cancer.^40,45^ As FTO inactivation impairs resistant cell growth,^40,45^ it represents a promising therapeutic target. Consequently, several inhibitors, such as the new generation FTO inhibitors FB23-2, CS1 and CS2,^46,47,86,87^ have been developed. All these inhibitors at least partially impair FTO enzymatic functions, suppress cancer cell growth *in vitro*, and reduce cancer burden in mice. In line with these findings, exposure of nilotinib-resistant K562 cells to CS1 promoted cell apoptosis (Fig. 3A, left) and inhibited cell proliferation (Fig. 3A, right). Since FTO knockdown was shown to enhance nilotinib sensitivity,^45^ we tested if CS1 could restore nilotinib sensitivity in resistant cells. As expected, the co-treatment of CS1 and nilotinib resulted in a more pronounced inhibition of cell proliferation (Fig. 3B, left) and promotion of cell apoptosis (46.8%) compared to single-agent treatment (control, 10.8%; nilotinib, 12.6%; FB23-2, 31.9%; Fig. 3B, right). The effects of CS1 were further verified upon exposure to FB23-2 (Fig. 3C, 3D). Exposure to the FTO inhibitors FB23-2, CS1, or CS2 led to a significant increase in FTO protein levels in K562, Kasumi-1, MV4–11, and SKNO-1 leukemia cells within 48 hours (Fig. 4). This finding is intriguing because the observed increase in FTO protein expression could decrease the therapeutic efficacy of these FTO inhibitors. Although the exact mechanism is not yet understood, this observation provides a strong justification for using FTO-specific siRNAs to reduce FTO protein expression, especially considering the potential off-target effects and toxicities of small-molecule FTO inhibitors.

### RNA m^6^A demethylation drives the high expression of LSC markers in TKI-resistant cells compared to TKI-sensitive cells

**Fig. 5.**
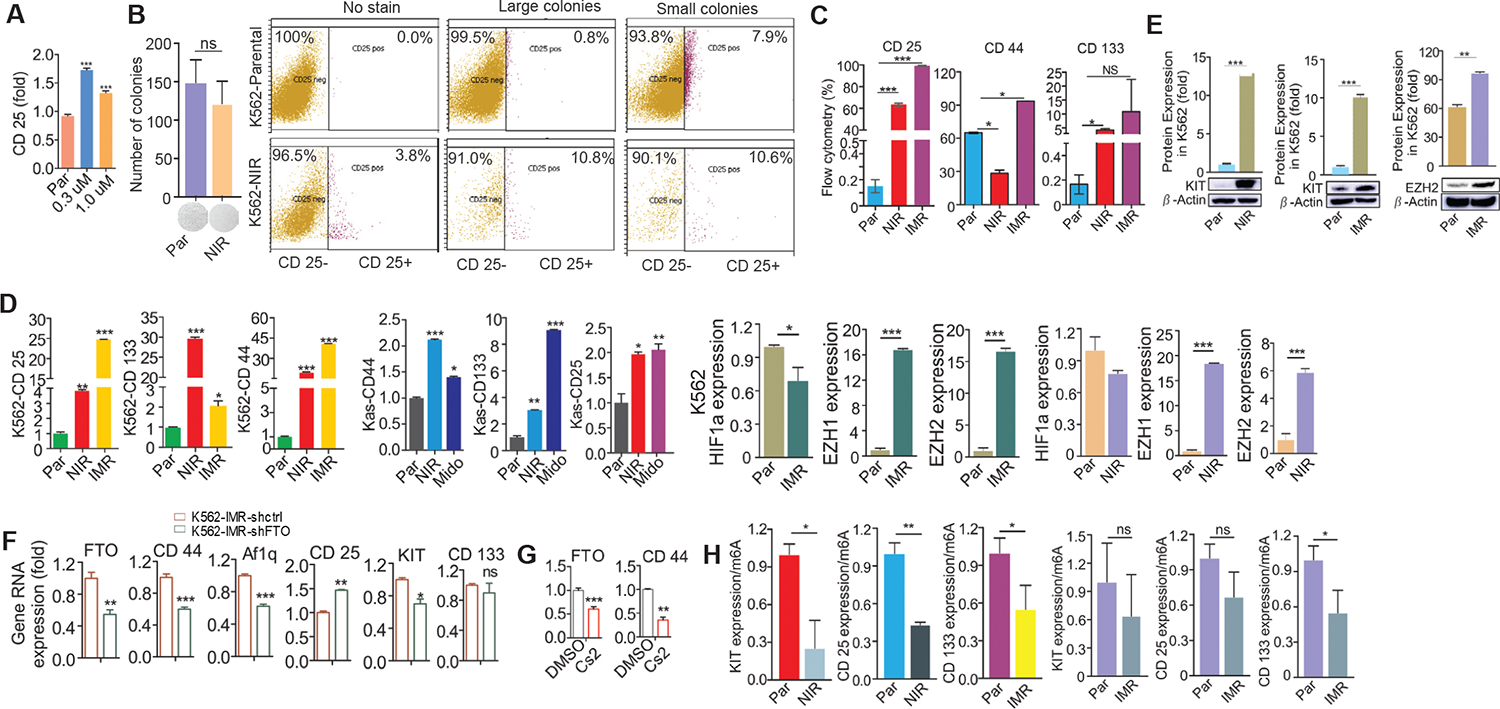
Expression and regulation of LSC markers in naïve, larger clones and TKI resistant leukemia cells. (A) Quantification of CD25+ cells in K562 cells treated with 0.3 or 1 μM of nilotinib for 48 hours, as measured by flow cytometry. (B) Left: Graphs representing colony numbers of parental and nilotinib-resistant K562 cells cultured in drug-free medium for 72 hours; Right: Flow cytometry analysis depicting the percentage of CD25+ cells in large vs small clones. (C) Graphs showing quantification of CD25+, CD44+ and CD133+ cells measured in parental vs NIR or IMR K562 cells, as measured by flow cytometry. (D) qPCR measuring indicated LSC marker expression in parental vs resistant Kasumi-1, MV4–11 and K562 cells. (E) Western blot and quantification of KIT (CD117) and EZH2 protein expression in parental, NIR-resistant, and IMR-resistant K562 cells. Band intensity was quantified and normalized to a loading control (β-actin). (F-G) qPCR measuring changes in the indicated stem cell markers in FTO knockdown (F) or FTO inhibitor CS2-treated (G) K562 cells resistant to nilotinib. (H) m^6^A immunoprecipitation (IP) was performed in mRNA from K562 resistant cells. The eluted RNA was converted to cDNA and transcript levels of KIT, CD25 and CD44 were measured by qPCR. Data are representative of three independent experiments. Par, parental; NIR, nilotinib resistance; IMR, imatinib resistance; Kas, Kasumi-1; shctrl, scramble control; *P <0.05, **P <0.01, ***P <0.001; ns, not statistically significant.

To further dissect the role of LSCs in TKI resistance, we measured the expression of CD117 (KIT), CD133, CD25 and CD44. These markers are commonly overexpressed in both AML and CML and are associated with TKI resistance.^78,79,88^ Specifically, their overexpression has been implicated in resistance to midostaurin and nilotinib in AML cell lines (Kasumi-1, MV4–11), as well as resistance to nilotinib and imatinib in the CML cell line K562. We also examined the expression of EZH1, EZH2 and HIF1α, all of which are known to significantly contribute to TKI resistance. Notably, nilotinib has also been utilized to treat AML, although less frequently than in BCR/ABL-positive CML and ALL.^45,80^ Flow cytometry analyses showed that K562 cells treated with nilotinib for 48 hours have a higher percentage of CD25+ cells (Fig. 5A). A greater proportion of CD25+ cells was also observed in the large (but not small) colonies of NIR cells compared to the parental K562 cells (Fig. 5B). While the total colony numbers were not significantly different between NIR and parental K562 cells (Fig. 5B, left), the percentage of CD25+ cells tended to be higher in large colony populations than small colonies (Fig. 5B, right) in the small colony populations. When compared to the parental K562 cells, both NIR and imatinib-resistant (IMR) cells showed a significantly higher percentage of cells expressing the markers CD25 and CD44. While the percentage of CD133+ cells was also significantly higher in NIR cells, the increase was only a non-significant trend in IMR cells (Fig. 5C). The elevated expressions of LSC cell surface markers CD25, CD133 and CD44, identified by flow cytometry, were further verified by qPCR in K562 cells resistant to nilotinib or imatinib and Kasumi-1 cells resistant to nilotinib or midostaurin/PKC412) (Fig. 5D). Western blot analysis further demonstrated the upregulation of KIT and EZH2 protein expression in K562 cells resistant to nilotinib or imatinib (Fig. 5E). Together, these findings support the premise that TKI exposure upregulates LSC markers at both the RNA and protein levels in leukemia cells.

To investigate how the expression of LSC markers is regulated in resistant cells, we first suppressed FTO activity using either shRNA knockdown or treatment with the inhibitor CS2. FTO knockdown by shRNA significantly reduces the RNA expression of CD44, Af1q and KIT; conversely, CD25 expression was upregulated, while CD133 showed only a trend toward downregulation (Fig. 5F). Surprisingly, while CS2 treatment also downregulated CD44, a LSC marker most strongly and consistently linked to TKI-resistant leukemia, it led to a decrease in FTO RNA expression (Fig. 5G). Considering that CS2 treatment increases FTO protein expression (Ref. Fig. 4), this suggests that as an m^6^A demethylase, the FTO may control its own gene expression via an m^6^A-mediated autoregulatory feedback loop. This mechanism merits further study in resistant leukemia. Next we examined m^6^A motifs ([G/A/U][G >A] m^6^AC[U >A/C]) within the transcripts of above LSC markers.^45,89,90^ The identification of m^6^A sites indicates that m^6^A mechanisms are likely involved in regulating these LSC markers. To test this, we performed m^6^A immunoprecipitation (IP) on mRNA from resistant and sensitive cells. After converting the eluted RNA to cDNA, we ran qPCR using primers covering the m^6^A motifs. Consistent with a global reduction of m^6^A in resistant cells,^45^ we found that m^6^A enrichment on KIT, CD25, and CD133 RNAs is significantly decreased in nilotinib-resistant cells and tends to be lower in imatinib-resistant cells (Fig. 5H). Notably, given that FTO is overexpressed and m^6^A enrichment on CD25 and CD133 RNAs is decreased in TKI-resistant cells,^45^ the findings that CD25 expression is increased and CD133 has a trend toward downregulation in FTO-knockdown implies that multiple m^6^A regulators may coordinately control CD25 or CD133 in resistant cells, which warrants further study. These results suggest that the FTO-m^6^A axis is a key regulator of LSC marker expression in TKI resistant cells.

### RNA nanoparticles with CD133 RNA aptamer show strong uptake in nilotinib-resistant cells and efficiently impair FTO expression

**Fig. 6.**
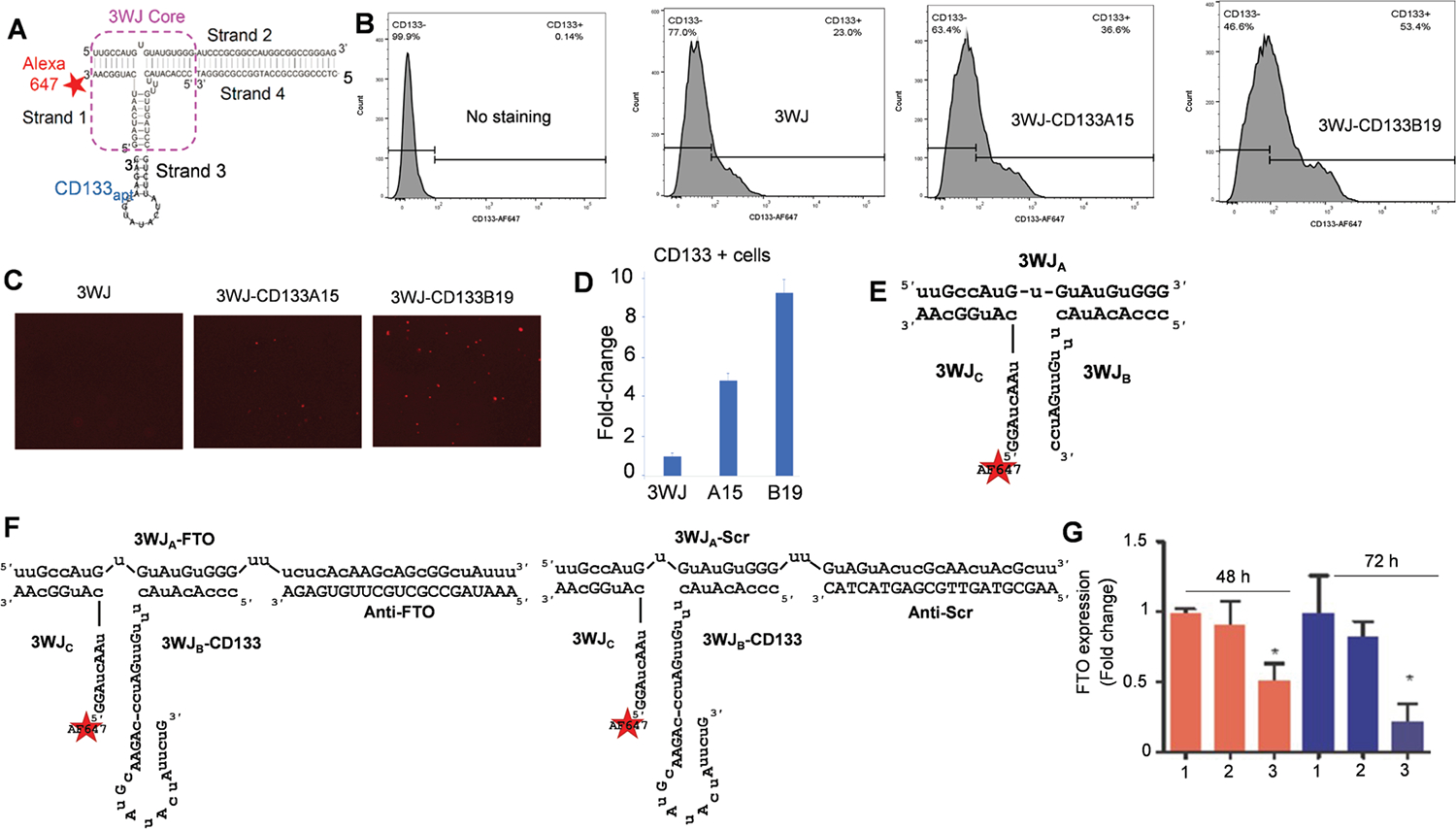
Strong uptake of CD133-targeted RNA nanoparticle and efficient knockdown of FTO in TKI resistant CML cells. (A) Design of 3WJ-CD133apt nanoparticles with Alexa-647 dye. (B-D) K562 resistant cells were stained by 50 nM of the indicated nanoparticles in FBS-free RPMI 1640 medium for 1 hour, and the labeled cells were detected by flow cytometry (B) and visualized by confocal microscope (C). Graphs are quantification of RNA nanoparticle-bound cells (D). Data represents three independent experiments. (E-F) Cartoon images showing the designs of RNA nanoparticles. (G) CD133-targeted RNA nanoparticles carrying *FTO* siRNA impair the expression of FTO gene in resistant CML cells. Imatinib resistant K562 cells were treated with 100 μM *FTO* siRNA-loaded RNA nanoparticles for 48 or 72 hours, and FTO expression was detected by qPCR. Data represents biological and technical duplicates. Note: 1, 3WJ; 2, 3WJ-CD133-siFTOScr-AF647; 3, 3WJ-CD133-siFTO-AF647; A15, 3WJ-CD133A15-AF647; B19, 3WJ-CD133B19-AF647; Scr, scramble; siR, siRNAs. *P <0.05.

Delivery of siRNAs to leukemia cells is challenging due to the lack of suitable cell surface markers. Because CD133 is important in developing TKI resistance,^78,79,88^ we hypothesized that the highly expressed CD133 in resistant K562 cells (ref. Fig. 5C, 5D) could serve as a drug delivery target. As proof of principle, we constructed and characterized RNA nanoparticles conjugated with CD133 RNA aptamers (A15, B19) (Fig. 6A). When exposed to these nanoparticles, resistant K562 cells (overexpressing CD133 and FTO) showed strong binding and uptake as detected by flow cytometry (Fig. 6B) and fluorescence microscopy. In these experiments, B19 consistently showed stronger binding (Fig. 6C, 6D). Overall, B19 demonstrated stronger binding than A15, suggesting it is the better RNA aptamer.

To inhibit resistant genes in TKI resistant cells, we targeted FTO, a mRNA m^6^A demethylase, because prior findings^40,45^ demonstrated that FTO overexpression enhances TKI resistance development, whereas FTO knockdown/inhibition impairs resistant cell growth, thus serving as a therapeutic target. Considering the pitfalls of existing strategies of FTO inactivation, including small molecule inhibitors and PROTACs,^46,49,50^ we then designed and constructed CD133-targeted RNA nanoparticles (B19) loaded with FTO siRNA (CRN-siFTO) (Fig. 6E, 6F). Of note, the negative controls were 3WJ motif that was derived from bacteriophage phi29 pRNA as a scaffold,^91^ adding CD133 RNA aptamer (B19)^92,93^ as targeting ligand, FTO siRNAs as therapeutic reagent and Alexa647 labeling for fluorescent imaging.

The sequences are: 3WJ-AF647: 3WJA: 5’-uuG ccA uGu GuA uGu GGG-3’; 3WJB: 5’- ccc AcA uAc uuu Guu GAu cc-3’; A647-3WJC: 5’-AF647- GGA ucA Auc AuG GcA A-3’; 3WJ-CD133-siFTO-AF647: A3WJ-FTO: 5’-uuG ccA uGu GuA uGu GGG uu ucu cAc AAG cAG cGG cuA uuu-3’; B-CD133: 5’-ccc AcA uAc uuu Guu GAu cc cAG AAc GuA uAc uAu ucu G-3’; C-AF647: 5’-AF647- GGA ucA Auc AuG GcA A-3’; Anti-FTO: 5’-AAA UAG CCG CUG CUU GUG AGA-3’; 3WJ- CD133-siScr-AF647: A3WJ-Scr: 5’-uuG ccA uGu GuA uGu GGG uu GuA GuA cuc GcA Acu AcG cuu-3’; B-CD133: 5’-ccc AcA uAc uuu Guu GAu cc cAG AAc GuA uAc uAu ucu G-3’; C-AF647: 5’-AF647-GGA ucA Auc AuG GcA A-3’; Anti-FTO: 5’-AAG CGT AGT TGC GAG TAC TAC-3’.

Exposure of resistant K562 cells to CRN-siFTO nanoparticles resulted in a notable impairment of FTO expression, with a significantly more pronounced knockdown observed at 72 hours compared to the effects of the vehicle and scrambled controls (Fig. 6G). This outcome implies that the CD133-targeting nanoparticles successfully deliver the siRNAs specifically to the resistant leukemia cells, thereby effectively inhibiting FTO expression.

### CD133-targeted nanoparticles containing FTO siRNA inhibit resistant cell growth

**Fig. 7.**
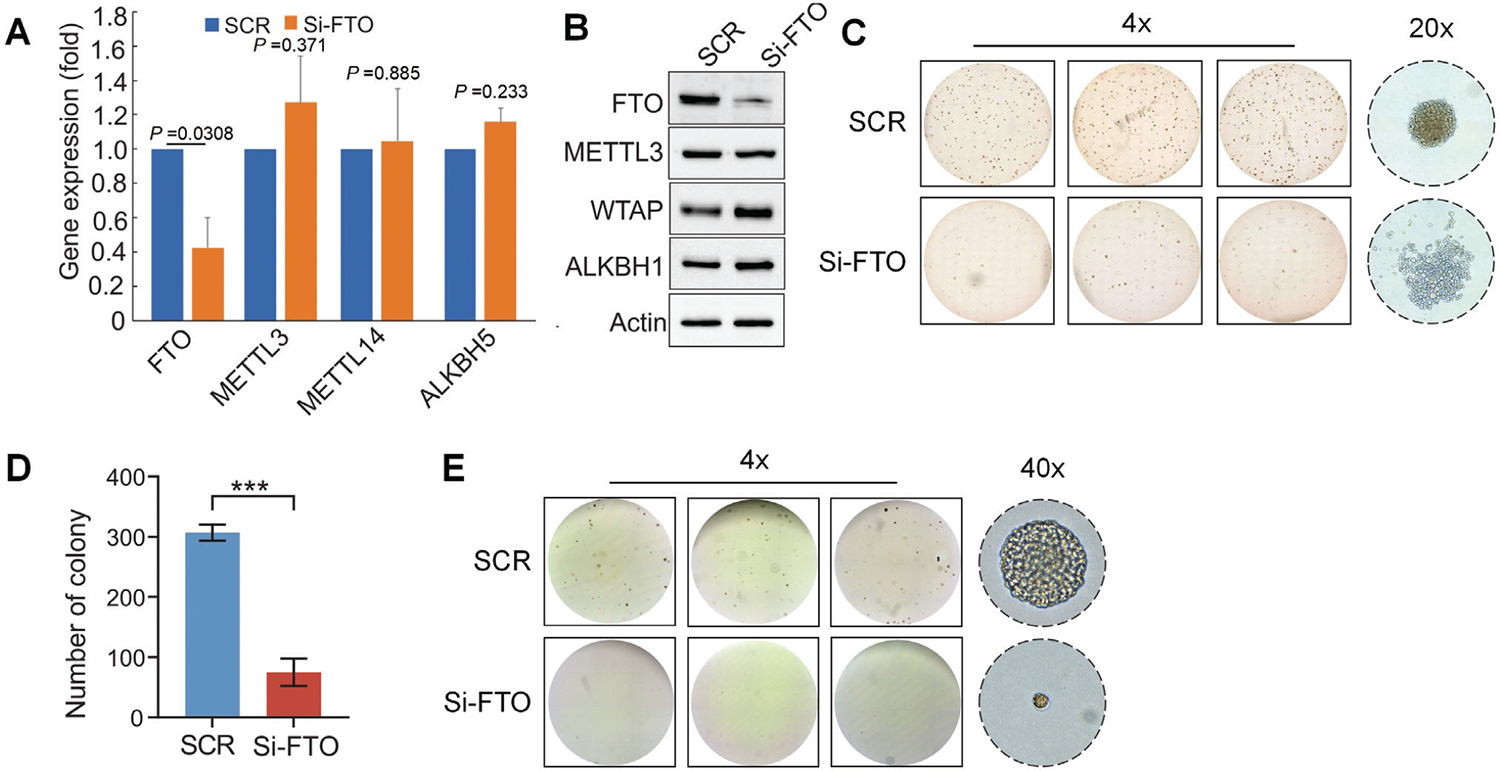
CD133-guided FTO siRNA nanoparticles inhibit growth in nilotinib-resistant K562 cells. Nilotinib-resistant K562 cells were treated with either 3WJ-CD133-siFTO-AF647 (si-FTO) or a control 3WJ-CD133-siFTOScr-AF647 (SCR). (A) qPCR analysis shows the expression of indicated m6A regulators at the RNA level. Data represents three independent biological replicates. (B) Western blot analysis shows the protein expression of the indicated m^6^A regulators. The blot shown is representative of triplicate drug treatment experiments. (C-D) Colony-forming analysis demonstrated the impaired proliferation of resistant cells post-treatment with si-FTO compared to SCR. C, the shown images are the overview of colony formation (4 ×; triplicate wells) and the representative colony (20 ×; right); D, graph is the quantification of colony number in three wells. The data shown is the average of triplicates with ±SEM. ***P <0.001. (E) Spheroid-forming assays reveal the inhibition of resistant cell growth by si-FTO compared to SCR. The shown images are the overview of spheroid formation (4 ×; triplicate wells) and the representative spheroids (40 ×; right).

Our previous studies demonstrated that FTO overexpression promotes resistant growth *in vitro* and tumor growth in mice.^45^ Based on our current findings that CD133-guided nanoparticles delivering FTO siRNA (CRN-siFTO) efficiently suppresses FTO expression (Ref. Fig. 6G), we investigated whether treatment with CRN-siFTO could overcome nilotinib resistance in K562 cells. To this end, nilotinib resistant K562 cells were exposed to 3WJ-CD133-siFTO-AF647 (CRN-siFTO) or 3WJ-CD133-siScr-AF647 (CRN-siScr). qPCR analysis and Western blot confirmed that CRN-siFTO treatment significantly decreases FTO RNA and protein expression compared to control CRN-siScr (Fig. 7A and 7B). The specificity of FTO knockdown was verified by the lack of changes in the expression of other m^6^A regulators, such as WTAP, METTL3, METTL14, ALKBH1 or ALKBH5. Functionally, colony- and spheroid-forming assays showed that the potential for colony and spheroid formation is significantly impaired, as evidenced by a reduction in sizes and number (Fig. 7C, 7D and 7E). These findings indicate that CD133-guided nanoparticles, used to deliver FTO siRNA, may be a promising strategy for overcoming TKI resistance. They also substantiate the essential role of FTO in acquired TKI resistance in leukemia.

## DISCUSSION

Drug delivery in leukemia is particularly challenging when compared to solid tumors, due to the absence of suitable surface markers and circulating nature of leukemia cells. In this study, we further delineated the molecular mechanisms underlying the development and sustenance of leukemia TKI resistance with a focus on leukemia stem cells, and exploited the effects of CD133-guided RNA nanoparticles containing FTO siRNAs on CML TKI resistant cells. Our comprehensive experimental and innovative findings document that the CD133-guided RNA nanoparticles carrying siRNAs for resistant gene FTO efficiently and specifically impair FTO expression in resistant cells overexpressing LSC marker CD133, leading to impairment of resistant cell growth. Our studies establish a customizable and scalable approach to use RNA nanotechnologies for overriding resistant leukemia cells.

Extending our previous discoveries^45^ that leukemia cells are epigenetically and phenotypically heterogeneous, we observed that naïve cell populations exhibit dynamic colony formation. Specifically, larger clones continued to grow, while smaller clones ultimately decayed and disappeared. We found that larger clones express higher levels of FTO with lower levels of FTO-dependent m^6^A methylation compared to smaller ones, and FTO expression is positively correlated with clone size.^45^ This disparity in clone sizes may contribute to drug tolerance in two ways. First, larger clones inherently possess a greater reservoir of pre-existing or readily adaptive drug-tolerant cells, increasing the statistical probability that a cell could acquire the necessary genetic mutation(s) for stable, heritable drug resistance. Second, while the higher drug tolerance in large naive clones is not, in itself, stable resistance, it may serve as a critical prerequisite for its development. During drug selection, most sensitive cells are eliminated, including the smaller clones with lower FTO and higher m^6^A expression.^45^ However, due to the dynamic and swiftly changeable nature of FTO/m^6^A, when exposed to TKIs, he larger clones—which initially have high FTO and low m^6^A—rapidly adjust their FTO/m^6^A levels. This dynamic change enables them to activate proliferation and anti-apoptotic genes, thereby surviving the drug treatment. Mechanistically, despite this phenotypic heterogeneity, FTO overexpression is consistently observed across the resistant population, even in resistant cells from leukemia patients. The resistance persists after both short-term and long-term withdrawal, albeit with partial resistance in the latter. Notably, FTO levels remained elevated throughout both withdrawal periods, highlighting the stable and persistent nature of FTO overexpression in these resistant cells. Our RNA nanoparticle treatment, performed during the short-term withdrawal when FTO is highly upregulated, confirmed the feasibility of our system for reducing FTO and inducing cell death in TKI-resistant populations. Furthermore, the ability of our nanoparticle to effectively deliver FTO siRNAs across this diverse cellular landscape suggests its potential to overcome the limitations of current therapies that target single resistance pathways. Further investigation into resistant blasts and leukemia mouse models is therefore warranted.

Compared to parental/sensitive cells, resistant populations, including CD44+ LSCs, form distinctive colonies and spheroids, supporting that TKI selection alters leukemia cellular plasticity. Another innovative finding is that LSC markers, including CD133, CD44 and KIT, are highly expressed in resistance vs parental cells. Not only does upregulation of these LSC markers substantiate the key contribution of LSCs to drug resistance, but also they serve as unique cell surface markers to guide drug delivery to specifically kill resistant leukemia cells. Indeed, resistant CML cells with CD133 upregulation show much higher cellular uptake of CD133-guided RNA nanoparticles compared to parental cells with lower CD133 expression. These findings offer a partial solution to the lack of available cell surface markers for developing targeted nanoparticle therapies in leukemia.

We previously showed^45^ that the FTO/m^6^A axis determines CML cell fate under drug selection through rapidly activating cell proliferative/anti-apoptotic genes. The present study revealed a new mechanism that FTO upregulation fuels TKI drug resistance by upregulating LSC markers, such as CD133, CD44 and KIT, and decreasing m^6^A abundance on RNAs of CD133, CD44 or KIT in resistant vs parental cells, which substantiates the role of the FTO-m^6^A axis in regulating LSC fate. These discoveries, together with the findings that FTO is highly expressed and plays an oncogenic role in leukemia and solid tumors^40,42,45,49,95^ support FTO as a new TKI resistance regulator, and a druggable target for overcoming TKI resistance. Indeed, leukemia cells with FTO overexpression have higher colony-forming potentials and lower sensitivity to TKIs, while FTO knockdown by siRNAs impairs colony formation and sensitizes TKI-resistant cells to TKI-induced cell death.^45^ In line, FTO inhibitors, such as FB23-2, CS1 and CS2^46,47,86,87^ to a greater degree, suppressed resistant cell proliferation and promoted cell apoptosis. Unexpectedly, the present study revealed an upregulation of FTO protein by FTO inhibitors, which may reduce their therapeutic effects. While FTO protein upregulation by FTO inhibitor treatment was unexpected, it is intriguing finding. This result does not contradict our model of a dysregulated FTO/m^6^A axis but rather points towards a potential compensatory feedback loop within the cellular regulatory machinery. A potential explanation is that as FTO’s enzymatic activity is inhibited, the cell may upregulate FTO protein expression to overcome the inhibition and restore normal m^6^A demethylation activity. While the specific mechanism may need further investigation, similar phenomena have been observed with other FTO inhibitors like 18097 that increases FTO protein levels in other cancer cell lines. Regardless, given that PROTACs for targeting FTO have the poor water solubility, low cellular permeability, and off-target side effects *in vivo*, and as the use of FTO inhibitory molecules increases FTO protein levels and causes severe toxicity and off-target effects,^46,49^ as well as the ability to deliver siRNAs to specific tissues at therapeutic doses *in vivo*, specifically for leukemia patients, remains a barrier, more efficient delivery strategies and tools are highly desirable.

Challenges have deterred the widespread use of RNA as a construction material, such as sensitivity to RNase degradation, susceptibility to dissociation after systemic injection, toxicity, and adverse immune responses. To date, these challenges have been largely overcome by 2’-fluoro (2’-F) or 2’-O-methyl (2’-OMe) modifications in the RNA that make it enzymatically stable in the serum, and improve thermal stability of the assembled RNA nanoparticles. The immunogenicity of RNA nanoparticles is sequence- and shape-dependent and is tunable.^69,96^ Such advantages make RNA nanotechnology an optimal platform to specifically and efficiently deliver oligos/drugs to tumors *in vivo*. The present study constructed RNA nanoparticles conjugating RNA aptamer of CD133 (an upregulated LSC marker in resistant cells) and carrying siRNAs for resistant gene FTO (overexpressed in resistant cells). As expected, resistant K562 cells have a much higher cellular uptake of these RNA nanoparticles due to CD133 overexpression. Concomitantly, FTO expression was efficiently impaired in treated vs untreated resistant cells. Given the previous findings showing that siRNAs-loaded RNA nanoparticles conjugated with EGFR RNA ligands induce tumor regression without obvious side effects and a reduction of healthy organ accumulation,^68,69^ the present study supports the feasibility of using the LSC marker (CD133) guided RNA nanoparticles containing siRNAs or/and inhibitors for FTO to overcome leukemia resistance.

Limitations of our study include: 1) testing only one LSC marker CD133. Given the upregulation of multiple LSC markers, such as CD44, CD25 or KIT, in resistant cells, the future studies should exploit additional markers as single or combination events to see which one is the most suitable marker for targeted delivery, and whether RNA nanoparticles conjugated two or more markers have larger binding affinity, more specificity and higher efficacy of drug delivery to inactivate target genes; 2) the absence of a control group using CD133-negative cells, although this study demonstrated the effect of CD133-guided nanoparticles. The goal is to examine the efficacy of nanoparticles in the same cells with and without CD133. Thus, a future study will use CRISPR-Cas9 to knock out CD133 expression in the resistant K562 cells. We will compare the binding and FTO knockdown efficacy of our CD133-guided nanoparticles between wild-type resistant K562 cells and the CD133-knockout resistant K562 cells. This experiment would provide definitive proof that the observed effect is dependent on CD133 expression; 3) examining only siRNAs. Other FTO inhibitory molecules (e.g., CS1/2, FB23-2) should be loaded together to achieve synergistic effects on FTO inactivation; 4) only *in vitro*. To increase the clinical implications, such RNA nanoparticles should be tested in *ex vivo* using resistant CML patient blasts, and *in vivo* using PDX (Patient-Derived Xenograft) or humanized mouse models bearing human CML resistant cells. However, such limitations are partially balanced by our mechanistic studies on the role of FTO/m^6^A in regulating LSCs and their markers, our characterization of CML cell phenotypic dynamics, and our development of first CD133-gauided RNA nanoparticles loading with FTO siRNAs in resistant leukemia.

## CONCLUSIONS

In conclusion, we have demonstrated the dynamic heterogeneity of naïve leukemia cells, which serves as an initial mechanism for resisting drug-induced cell death. Our findings identified an upregulation of LSC markers driven by an FTO-m^6^A pathway. This deepens our understanding of cancer drug resistance and presents new cell-surface markers for targeted drug delivery in leukemia. By leveraging the elevated expression of CD133 on resistant cells, our studies established CD133-guided RNA nanoparticles carrying FTO siRNAs as a novel therapeutic option, particularly for patients with relapsed leukemia.

## EXPERIMENTAL METHODS

### Cell lines and cell culture

Leukemia cell lines, K562, MV4–11 and Kasumi-1, were newly purchased from American Type Culture Collection with no further authentication or testing for mycoplasma. SKNO-1 cell line was kindly provided by Dr. Clara Nervi (University of Rome “La Sapienza”). The K562 is a human CML cell line that was derived from CML patients in blast crisis. K562 cells are erythroleukemia type, non-adherent and rounded and positive for the *BCR::ABL1* fusion gene. K562 cells lack the MHC complex and develop characteristics like early-stage erythrocytes, granulocytes and monocytes; the MV4–11, established from the blast cells of male child with biphenotypic B-myelomonocytic leukemia, is a human AML cell line, non-adherent and carry translocation t(4;11) (*MLL-AF4* fusion gene) and a FLT3-ITD (FLT3 internal tandem duplications) mutation; the Kasumi-1, isolated from the peripheral blood of an Asian male patient with AML, is a human AML cell line carrying translocation t(8;21) (*AML1/ETO* fusion gene) and c-KIT activating mutation. Kasumi-1 cells have the characteristics of myeloid and macrophage lineages; the SKNO-1 cell line is a human myeloid leukemia cell line established from the bone marrow of a patient with AML (M2). SKNO-1 cells carry translocation t(8;21) (AML1/ETO fusion gene) and c-KIT activating mutation. Cell lines were grown in RPMI-1640 (GE Healthcare #SH30027.01) supplemented with 20% (Kasumi-1, SKNO-1) or 10% (K562, MV4–11) fetal bovine serum (FBS, Gibco by Life Technologies^™^ #16140–071) and Antibiotic-Antimycotic (Gibco by Life Technologies^™^ #15240062) at 37°C under 5% CO_2_. No cell line used in this paper is listed in the database of commonly misidentified cell lines maintained by ICLAC (International Cell Line Authentication Committee).

### Plasmid design and construction

Three shRNAs against *FTO* (TRCN0000183897, TRCN0000179651, TRCN0000180978) and the negative control vectors (pLKO.1) were obtained from BMGC RNAi (University of Minnesota).

### *In vitro* adaptation of TKI resistant cells

Cell lines, K562, Kasumi-1 and MV4–11, were passaged with low concentration of imatinib or nilotinib (0.1 μM) and sequentially cultured in increasing concentrations of these TKIs (0.3, 1 μM) for 8–10 weeks. Cells cultured in parallel in drug-free medium were used as parental/sensitive controls. Cells were considered resistant when they could routinely grow in medium containing 1 μM imatinib, or nilotinib, respectively. The cells were maintained in medium containing 1 μM imatinib or nilotinib. For further investigations, the above leukemia parental and TKI resistant cells were washed once by blank RPMI1640 medium (no FBS, no antibiotics) to remove any residual TKIs. The washed cells were cultured in regular TKI-free RPMI 1640 medium (containing 10% FBS, antibiotics) for 48–72 hours to allow for recovery and proliferation.

### Lentivirus vector, virus production and virus infection

For virus production, HEK-293 (3.8 × 10^6^) cells were planted in a 10 cm cell culture dish for 24 hours, and transfected with 6 μg of target or scrambled control plasmids using calcium phosphate transfection reagent (CalPhos^™^ Mammalian Transfection Kit), following the manufacture’s instruction. The lentiviruses were harvested at 48 and 72 hours after transfection and concentrated using the protocol of the Lenti-X^™^ Concentrator (Clotech #631232). For virus infection, leukemia cells (1 × 10^6^) were infected by the lentiviruses using Polybrene (final concentrate 4 μg/ml) in 1ml medium and Puromycin (final concentration 2 μg/ml) was added to select the stable transformants 24 hours post-infection.

### Design, construction and characterization of RNA nanoparticles

Based on published methods,^68,91^ we constructed and characterized FTO siRNA-loaded RNA nanoparticles conjugated with CD133.^68^ A single steroid molecule, cholesterol-triethylene glycol, was conjugated into the tail of the pRNA-3WJ to promote anchorage of 3WJ onto the EV membrane.^91,97,98^ The cholesterol-modified RNA nanoparticles were incubated with EVs at 37°C for 1 hour to connect RNA nanoparticles to the EV surface. To enhance targeting TKI-resistant cells, a B19 CD133 RNA aptamer was incorporated at one end of arrowtail pRNA-3WJ and thereby displayed on EVs. One pRNA-3WJ strand was end-labelled with Alexa647. To improve the stability of siRNAs *in vivo*, all RNA oligos were 2′-F-modified on pyrimidines to provide RNase resistance; the guide strands of siRNAs were unmodified. For tracking siRNA loading efficiency in EVs, the FTO siRNAs were fused to an Alexa647-labelled 3WJ core and assembled into RNA nanoparticles. The groups are 3WJ-EVs only, 3WJ-CD133-EVs, 3WJ-EVs/scramble, 3WJ-CD133-EVs/scramble, 3WJ-EVs/FTOsiRNA, 3WJ-CD133-EVs/FTOsiRNA.

### Transfection of K562 resistant cells with RNA nanoparticles

K562 resistant cells were washed once by blank RPMI1640 medium (no FBS, no antibiotics) to remove any residual TKIs. The washed cells were cultured in fresh, complete RPMI 1640 medium (containing 10% FBS, antibiotics) for 48 hours to allow for recovery and proliferation. Following the 48-hour incubation, the cells were washed once with pre-warmed, blank RPMI 1640 medium to remove FBS and antibiotics. The nanoparticle staining solution was prepared by adding 2.5 μl of a 10 μM nanoparticle stock solution to 500 μl of blank RPMI 1640 medium, resulting in a final concentration of 50 nM. The cell medium was replaced with the 500 μl nanoparticle solution, and the cells were incubated with nanoparticles for 1 hour at 37°C to allow for staining. The nanoparticle groups include: 3WJ-Alexa647 (Ve), 3WJ-CD133A15-Alexa647, 3WJ-CD133B19-Alexa647, 3WJ-CD133B19-siScr-Alexa647 and 3WJ-CD133B19-siFTO-Alexa647. The stained cells were subjected to Flow and florescent microscope assays.

### Characterization of resistant and parental cells by magnetic or flow cell sorting

Leukemia parental and resistant cells were incubated with microbeads coated by antibody against CD44 (CD44 MicroBeads, Miltenyi Biotec, 130-095-194). The respective positive cells were isolated using L3T4 microbeads and MACS LS columns (Miltenyi Biotec) as manufacturer’s recommendations. To measure the percentage of LSCs, different TKI resistant and parental cells were stained with antibodies against CD44 (Miltenyi Biotec, 130-113-334), CD25 (Miltenyi Biotec, 170-081-060) or CD133 (Miltenyi Biotec, 170-081-001).

### Colony-forming assays

Colony-forming assays were performed using MethoCult^®^ medium (Stem Cell Technologies #03434) as previously reported.^45,81,99^ Briefly, the cells were suspended in 0.3 ml of IMDM medium (Stem Cell Technologies #36150), mixed with 3 ml MethoCult^®^ medium and then dispensed into 35 mm dishes. The colony count and size were recorded after 7–10 days. Generally, 10 single clones were picked up into 96 well plates per each cell line to get the stable clones after virus infection and puromycin selection.

### Cell proliferation and apoptosis assays

Cell proliferation assays were performed using Cell Counting Kit-8 (CCK-8, Dojindo Molecular Technologies #CK04–11) as previously reported.^80,81,100^ Briefly, the parental and resistant cells with various treatment (1.5 × 10^4^) in RPMI-1640 medium (100 μl) were dispensed into 96-well flat-bottomed microplates and incubated for 24 hours. The cells were cultured for another 24 or 48 hours, and the CCK-8 reagent (10 μl) was added to each well. The microplates were incubated at 37°C for an additional 2~4 hours. Absorbance was read at 450 nm using a microplate reader and the results were expressed as a ratio of the treated over untreated cells (as 100%). Five wells were sampled per experimental group in each experiment. Averages are reported ±SD. Cell apoptosis assays were performed using Annexin V-PI Apoptosis Detection Kit I (BD Pharmingen^™^ #556547) according to the manufacturer’s instruction and followed by flow cytometry analysis.

### 3D Spheroid-forming assays

Spheroid assays were performed using established protocols.^101,102^ Briefly, 500 cells are suspended in 20 μL medium plus 0.25% methylcellulose (v/v), and seeded as a single hanging drop, on the cover of 4-well culture plates. Drops are kept in standard culture conditions for 3–15 days. Spheroids are imaged using a Zeiss i880 confocal/multiphoton microscope. 2-photon excitation is done with 800 nm light, and the fluorophores are separated by emission.

### Western blotting

The whole cellular lysates were prepared by harvesting the cells in 1 × cell lysis buffer [20 mM HEPES (pH 7.0), 150 mM NaCl and 0.1% NP40] supplemented with 1 mM phenylmethane sulfonyl fluoride (PMSF, Sigma #10837091001), 1 × Phosphatase Inhibitor Cocktail 2 and 3 (Sigma #P5726, P0044), and 1 × protease inhibitors (protease inhibitor cocktail set III, Calbiochem-Novabiochem #539134). The proteins were resolved by sodium dodecyl sulfate (SDS)–polyacrylamide gel electrophoresis, transferred onto PVDF membranes (GE Healthcare #10600023), blocked by 5% non-fat milk followed by probing with first (anti-FTO, AdipoGen, AG-20A-0064; anti-KIT, Abcam, #ab32363; anti-CD44, Abcam, ab243894; anti-CD25, Abcam, #ab231441; anti-AF1q, Abcam, ab109016) and HRP-conjugated secondary (goat anti-rabbit IgG, Cell Signaling, 7076S) antibodies.

### RNA isolation, cDNA preparation and quantitative PCR (qPCR)

According to manufacturers’ instructions, the total RNA was isolated using miRNeasy Kit (Qiaqen #217004), and complementary DNA (cDNA) synthesis was performed using SuperScript^®^ III First-Strand Synthesis System (Invitrogen #18080-051). The expression of target genes was assessed by SYBR Green qPCR (Applied Biosystems #4309155) and normalized by GAPDH levels. The primers are:

KIT: F 5’-GTCTCCACCATCCATCCATCC-3’, R 5’-CACGTGTATTTGCCGGTGTT-3’;
CD25: F 5’-CTGCAGAGAAAGACCTCCGC-3’, R 5’GGACGAGTGGCTAGAGTTTCC-3’;
CD44: F 5’-AACCCTTGCAACATTGCCTGA-3’, R 5’-GCTTCCAGAGTTACGCCCTTGA-3’;
CD133: F 5’-GGAGGCACCAAGTTCTACCT-3’, R 5’-CGCGGCTGTACCACATAGAG-3’;
AF1q: F 5’-GGACCCTGTGAGTAGCCAGTA-3’, R 5’-CTTGCCCGATCATTTTGCCA-3’;
GAPDH: F 5’- AATGAAGGGGTCATTGATGG-3’, R 5’-AAGGTGAAGGTCGGAGTCAA-3’.

### m^6^A immunoprecipitation (IP)

The RNAs were diluted in 200 μl IPP buffer (150 mM NaCl, 0.1% NP-40, 10 mM Tris-HCl, pH 7.4) and fragmented into 100-nucleotide-long fragments using sonication. The fragmented RNAs were incubated for 12 hours at 4 °C with 5 μl anti-m^6^A (#202003, Synaptic System) in IPP buffer. The mixture was then immunoprecipitated by incubation with Dynabeads^™^ Protein G (ThermoFisher #10004D) at 4 °C for additional 3 hours. After extensive washing by IPP buffer, 75 μl 42°C pre-heated Elution Buffer (0.02 M DTT, 0.15 M NaCl, 0.05 M Tris-HCl, pH 7.4, 0.001 M EDTA, 0.1% SDS) were added into m^6^A-positive RNA solution for 5 min at 42°C, and this step was repeated 2 times. Finally, the enriched m^6^A RNA was eluted from the beads into 225 μl solution and precipitated by adding 2.5 times volume of 100% ethanol.

### Statistical analysis

The statistical analysis was performed using the Student’s t-test, and if the variances are not equal (or not homogeneous), a non-parametric test is adopted. All analyses were performed using the GraphPad Prism 5 Software. *P* <0.05 was considered statistically significant. All *P* values were two-tailed. No blinding or randomization was used. All criteria were pre-established. No statistical method was used to predetermine sample size and the sample size for all experiments was not chosen with consideration of adequate power to detect a pre-specified effect size. Variations were compatible between groups. *In vitro* experiments, such as qPCR, Western blotting and cell proliferation assays were routinely repeated three times unless indicated otherwise in Figure legends or main text. For every Figure, the statistical tests were justified as appropriate.
